# Reduction of phenolics in faba bean meal using recombinantly produced and purified *Bacillus ligniniphilus* catechol 2,3-dioxygenase

**DOI:** 10.1186/s40643-023-00633-8

**Published:** 2023-02-12

**Authors:** Rebecca M. Murphy, Joanna C. Stanczyk, Fang Huang, Matthew E. Loewen, Trent C. Yang, Michele C. Loewen

**Affiliations:** 1 Department of Chemistry and Biomolecular Sciences, University of Ottawa, 150 Louis-Pasteur Pvt, Ottawa, ON K1N 6N5 Canada; 2 Aquatic and Crop Resources Development Research Center, National Research Council of Canada, 100 Sussex Drive, Ottawa, ON K1A 0R6 Canada; 3 Department of Veterinary Biomedical Sciences, Western College of Veterinary Medicine, University of Saskatchewan, 52 Campus Drive, Saskatoon, SK S7N 5B4 Canada

**Keywords:** Pulse meal, *Vicia faba*, Biocatalysis, Catechol 2,3 dioxygenase, Phenol reduction

## Abstract

Pulse meal should be a valuable product in the animal feed industry based on its strong nutritional and protein profiles. However, it contains anti-nutritional compounds including phenolics (large and small molecular weight), which must be addressed to increase uptake by the industry. Microbial fermentation is currently used as a strategy to decrease larger molecular weight poly-phenolics, but results in the undesirable accumulation of small mono-phenolics. Here, we investigate cell-free biocatalytic reduction of phenolic content in faba bean (*Vicia faba* L.) meal. A representative phenolic ring-breaking catechol dioxygenase, *Bacillus ligniniphilus* L1 catechol 2,3-dioxygenase (BLC23O) was used in this proof-of concept based on its known stability and broad substrate specificity. Initially, large-scale fermentative recombinant production and purification of BLC23O was carried out, with functionality validated by in vitro kinetic analysis. When applied to faba bean meal, BLC23O yielded greatest reductions in phenolic content in a coarse air classified fraction (high carbohydrate), compared to either a fine fraction (high protein) or the original unfractionated meal. However, the upstream hydrolytic release of phenolics from higher molecular weight species (e.g. tannins, or complexes with proteins and carbohydrates) likely remains a rate limiting step, in the absence of other enzymes or microbial fermentation. Consistent with this, when applied to a selection of commercially available purified phenolic compounds, known to occur in faba bean, BLC23O was found to have high activity against monophenolic acids and little if any detectable activity against larger molecular weight compounds. Overall, this study highlights the potential viability of the biocatalytic processing of pulse meals, for optimization of their nutritional and economical value in the animal feed industry.

**Graphical Abstract:**

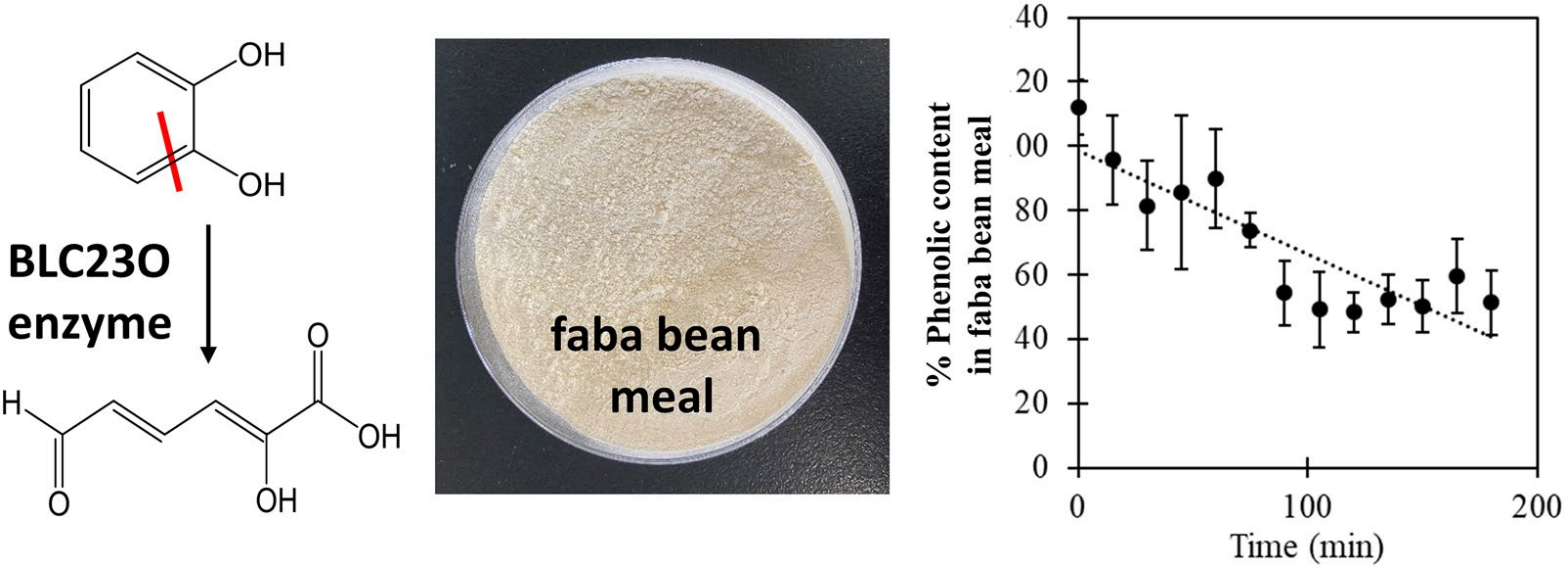

**Supplementary Information:**

The online version contains supplementary material available at 10.1186/s40643-023-00633-8.

## Introduction

Alternative feed ingredients and innovation of feed technology are progressively being investigated and incorporated into processing pipelines toward enhanced digestibility and efficiency, decreased environmental impact and maximized economic opportunity (Behnke 1996; der Poel et al. 2020).

Pulses (*Fabaceae* family) and their by-products are examples of advantageous sources of animal feed that could be further optimized to increase their value and applicability (Sherasia et al. 2018; Singh 2017). Currently, pulses play an important role in sustainable development based on their ability to fix nitrogen, reducing the need for costly nitrogen fertilizers when used in crop rotations, and sequestering greenhouse gas from the environment (Irisarri et al. 2021). Furthermore, pulse nutrient and protein-rich profiles significantly furthers their potential. For example, pulse meal, a by-product of the industry, is approximately 40–45% protein on a dry mass basis and thus holds great potential to serve as animal feed, contributing to food security and reducing competition with human food (Sherasia et al. 2018). Such applications of by-products also reduce costs related to disposal and allow for the conversion of low value products into higher value feed/food (Ominski et al. 2021).

Phenolics (e.g. tannins, anthocyanins, flavones, flavonols, flavanones, benzoic acids, cinnamic acids, etc.) are intrinsic to pulses, and in the context of animal nutrition, are considered anti-nutritional factors (ANFs) based on observed negative effects on digestion and the bioavailability of nutrients (Kardum and Glibetic 2018; Kumar et al. 2021). These effects are due to phenolics forming complexes with storage proteins, interfering with peptide bond proteolysis, and serving as direct inhibitors of digestive enzymes, together decreasing protein and carbohydrate availability (Morzel et al. 2022; Punia et al. 2021). Chelation of metal ions by poly-phenolics has also been shown to reduce absorption and decrease uptake of vitamins and minerals (Singh 2017). Finally, the palatability of pulse meal is reduced by tannins in particular, due to undesirable astringency, which lowers food intake and animal performance (Singh 2017). Thus, reducing phenol content in pulse products remains an important goal for increased nutritional and economic value.

**Fig. 1:**
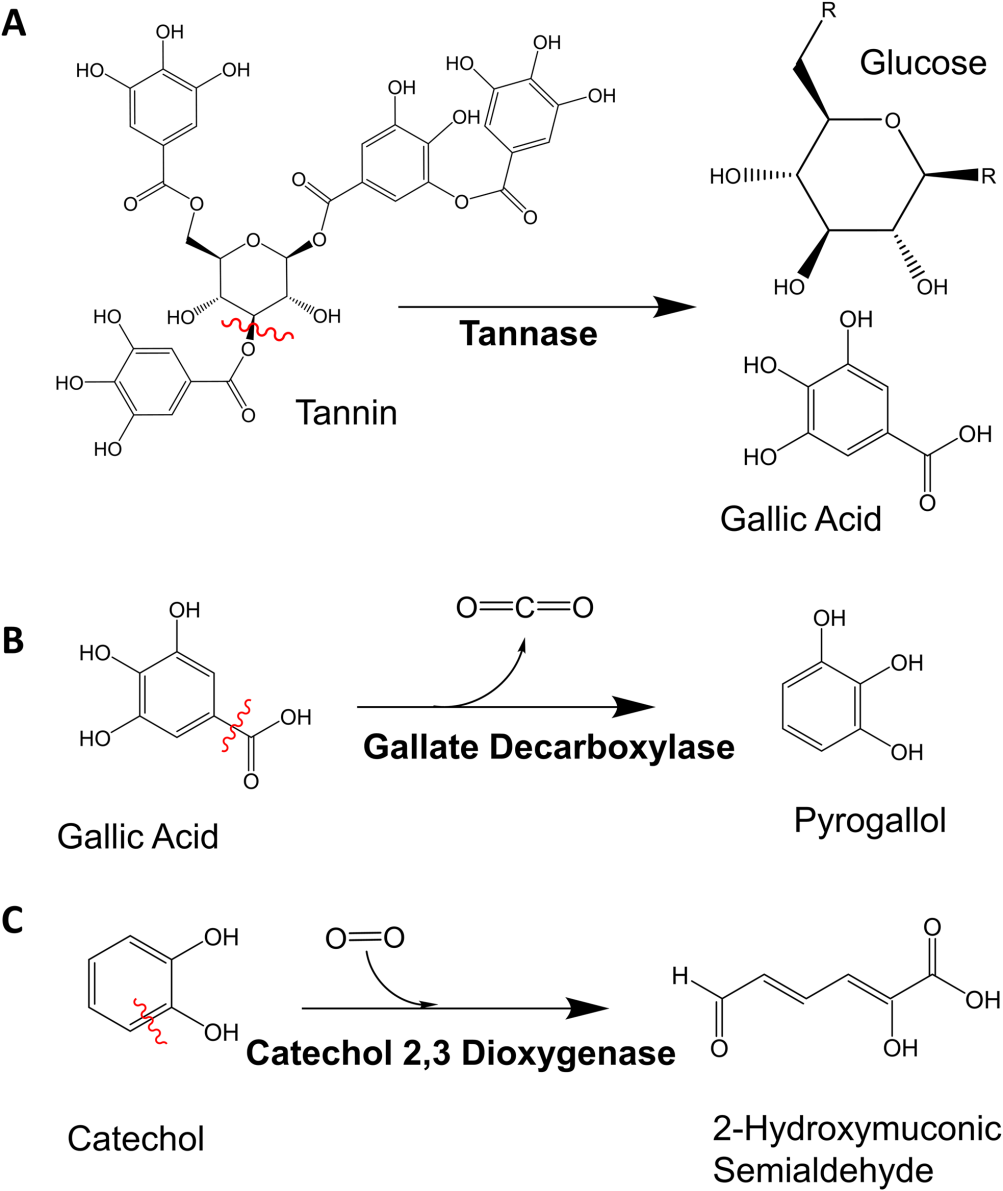
Enzymatic reactions showing substrates and products relevant to this study.** A** tannase, **B** gallate decarboxylase, **C** catechol 2,3 dioxygenase

Currently, microbial fermentation is used as a strategy to lower ANFs in feeds (der Poel et al. 2020; Olukomaiya et al. 2019). For example, *Lactobacillus plantarum*, a Gram-positive lactic acid bacterium commonly used in the food industry, specifically possesses genes encoding tannin acyl hydrolase (TAH, commonly known as tannase) and gallate decarboxylase, together yielding the mono-phenol pyrogallol (Jimenez et al. 2014). TAH belongs to the superfamily of esterases that hydrolyze the ester and depside bonds in gallic acid tannins or hydrolyzable tannins, leading to the release of gallic acid and glucose (Fig. 1A; (Govindarajan et al. 2016)). Gallate decarboxylase a member of the carboxy-lyase superfamily, catalyses the non-hydrolytic removal of the carboxylic group from the ring yielding pyrogallol and carbon dioxide (Fig. 1B; (Jimenez et al. 2013)).

However, the observed accumulation of mono-phenols such as pyrogallol in *L. plantarum* fermented feeds is also undesirable, with these small molecular weight compounds eliciting similar anti-nutritional effects as the higher molecular weight compounds (Kardum and Glibetic 2018; Kumar et al. 2021). The next step in degradation of mono-phenols generally involves breaking the phenolic ring. In nature, this activity relies on a family of microbial catechol dioxygenases that carry out the oxidative cleavage of hydroxylated aromatic rings, to yield linear compounds that feed into the citric acid cycle (Fig. 1C; (Kamimura et al. 2017)). Phenolic ring cleavage can be intradiol (between two consecutive hydroxyl groups on the ring (positions 1 and 2)), proximal extradiol (next to the hydroxyls; position 2 and 3) or distal extradiol (removed from the hydroxyls; position 4 and 5) (Hou et al. 1977). A brief review of the current literature and a search of the NCBI database suggests *L. plantarum* does not encode any members of this family of enzymes, consistent with the high accumulation of phenolics in such fermentations. Thus, as an alternative or complement to microbial fermentation, cell-free biocatalysis has been proposed for reduction of phenolic content. A biocatalytic strategy would decouple tannin and phenolic degradation from cellular physiology, reducing any downregulation imposed by the carbohydrate-rich environment and feedback inhibition, as well as allowing absolute control of enzyme types and concentrations applied (Claassens et al. 2019).

One potential candidate for cell-free phenolic degradation of pulse meal is a *Bacillus ligniniphilus* L1 catechol 2,3-dioxygenase (BLC23O; (Adewale et al. 2021)). *B. ligniniphilus* L1 is a halotolerant and alkaliphilic bacterium isolated from sediments from the South China Sea, known to use poly-phenolic lignin as its sole carbon source. Catechol 2,3-dioxygenases elicit proximal extradiol cleavage. Three catechol 2,3-dioxygenases encoded by *B. ligniniphilus* L1 have been identified and the shortest one, a protein of 283 amino acids (BLC23O, NCBI accession WP_017726464.1) with a calculated molecular mass of approximately 32 kDa, was recently characterized (Adewale et al. 2021). This enzyme was found to have unusually broad substrate specificity and good thermostability, traits proposed to be associated with its atypical monomeric structure.

Here the application of BLC23O to cell-free reduction of phenolic content in faba bean meal is assessed. Scaled-up recombinant production and purification of the enzyme are described, followed by evaluation of the enzyme’s potential to reduce phenolics in three different meal fractions from *Vicia faba* L. (faba bean) (Martineau-Cote et al. 2022) and in an array of commercially available standard phenolics known to be present in faba bean.

## Results

### Large-scale (4-L fermentative) production and Ni–NTA enrichment of BLC23O

**Fig. 2:**
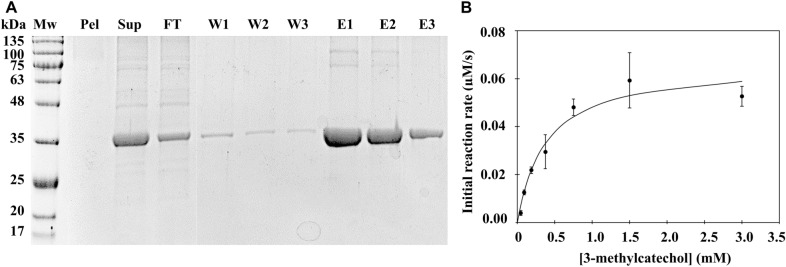
Purification and characterization of recombinant BLC23O. **A** Ni–NTA Affinity purification of recombinant BLC23O. SDS–PAGE showing the obtained pellet (P) and supernatant (S) fractions from an *E. coli* lysate. Subsequent flow-through (FT), washes (W) and elutions (E) are included. The elution fractions were pooled and used in all subsequent experiments. A small portion of the pooled elution was further purified by SEC (Additional file 1: Figure S1). **B** Kinetic analysis of Ni–NTA + SEC purified BLC23O. Following SEC purification, a Michaelis–Menten kinetic analysis of BLC23O catalytic activity was conducted. BLC23O (18.7 µg/mL) was combined with 10 mM of the co-factor Mn^2+^ and varying concentrations of the substrate 3-methylcatechol. The data were fitted to the Michaelis–Menten equation, using Microsoft Excel’s Solver

Shake flask production of BLC23O from *Escherichia coli* has been previously reported (Adewale et al. 2021). Here production of BLC23O was scaled up to a 4-L fermentative format. Wet weight determination of the obtained cell pellet was 49.8 g, or 12.4 g cell pellet/L of culture. Outcomes of the affinity-based enrichment of the recombinant His-tagged BLC23O are shown in Fig. 2A. Thick bands at the expected Mw of approximately 35 kDa are present in the three elution fractions and the original supernatant fraction. Small amounts of protein were also detected at 35 kDa in the washes and flow-through, suggesting minor losses during the enrichment. No BLC23O was detected in the lysis pellet, indicating that cell lysis was successful. Contaminating proteins were largely eliminated from the final sample. The elution fractions were pooled and protein quantified, yielding 116 mg of enzyme/L of cell culture, or 9.3 mg of BLC23O / g of wet weight cells.

### Kinetic analysis of BLC23O

A small portion (4%) of the obtained protein from Ni–NTA enrichment was subjected to further purification by size-exclusion chromatography. The chromatographic separation curve for BLC23O (Additional file 1: Figure S1A) showed a clear and steep peak at 82 min, consistent with the expected molecular weight of 35 kDa upon calibration (Additional file 1: Figure S1B). Collected fractions spanning this peak were visualized by SDS-PAGE, showing a single band at the expected Mw of 35 kDa (Additional file 1: Figure S1C). A Michaelis–Menten analysis of the obtained purified BLC23O against varying concentrations of the known substrate, 3-methylcatechol, yielded a K_M_ of 379 ± 86 µM, a turnover number (kcat) of 0.13 ± 0.01 s^−1^, and a catalytic efficiency (kcat/K_M_) of 340 ± 80 M^−1^ s^−1^ (Fig. 2B). Comparison of these results with a previous kinetic analysis for BLC23O highlights non-significant differences (previously: K_M_ 418 ± 25 µM, kcat 0.20 ± 0.03 s^−1^ and kcat/K_M_ 480 ± 80 M^−1^ s^−1^; (Adewale et al. 2021)).

### Measurement of phenolic content in faba bean meal

**Fig. 3:**
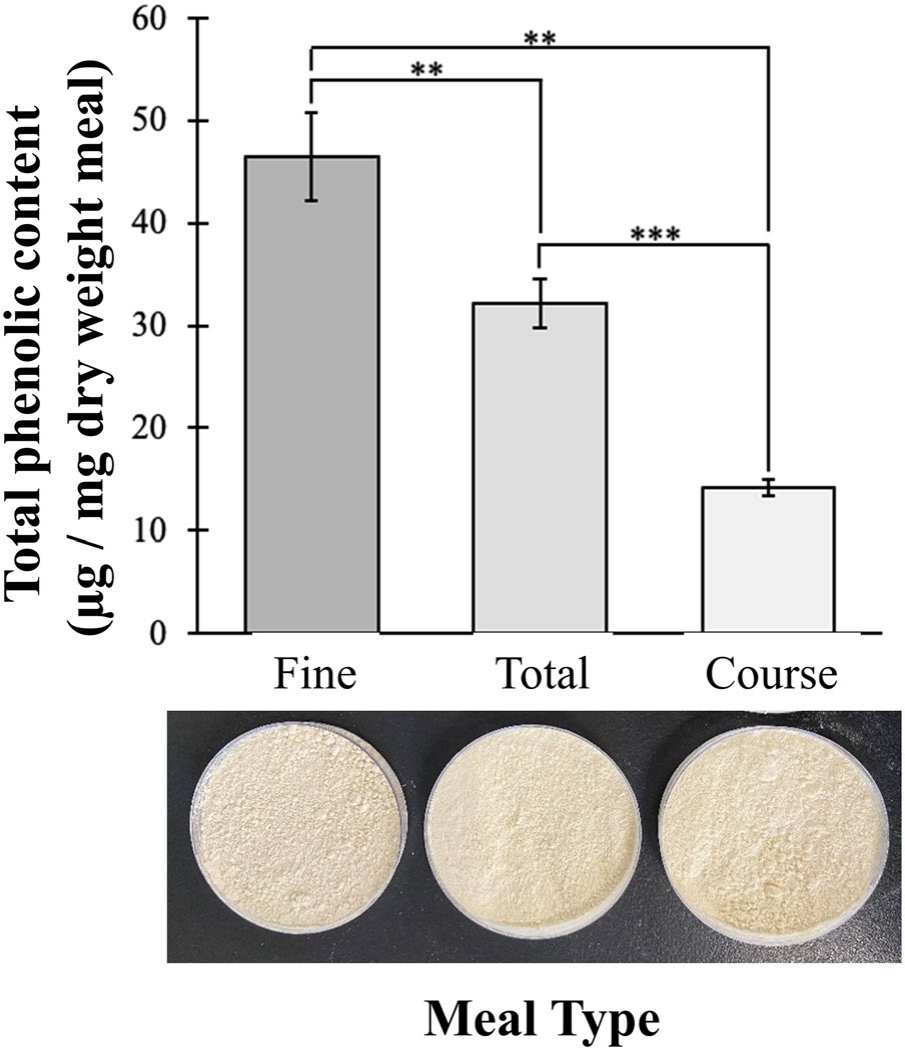
Total phenolic content of three faba bean meal fractions. Fine, total, and coarse faba bean meal fractions (see representative images) were reacted with the Folin–Ciocalteu reagent for 45 min. The absorbance at 725 nm was obtained and compared to a calibration curve for tannic acid (Additional file 1: Figure S2). Total phenolic content is represented as µg of phenolics per mg (dry weight) meal. Data are presented as the mean and standard deviation of the results (*n* = 3). **p < 0.01, ***p < *0.001*

Initially, the phenolic content in three distinct samples of faba bean meal was assessed, including the original ‘total’ meal, as well as ‘fine’ and ‘coarse’ fractions of the meal obtained from air-classification. Analysis of the phenolic content of each fraction was completed using the Folin–Ciocalteu (F–C; Blainski et al. 2013; Everette et al. 2010)) reagent with calibration based on tannic acid (Fig. 3 and Additional file 1: Figure S2A). Statistically significant differences in phenolic content were detected, where the fine fraction had the highest total phenolic content, followed by total meal, and then the coarse fraction. The kinetics of the F–C reagent were evaluated in more detail at the maximum amount of phenolics detected in meal reactions, using 14 µg/mL tannic acid (Additional file 1: Figure S2B). Under these conditions the reaction was complete within 60–70 min and phenolic content remained constant for an additional 120 min thereafter. These outcomes highlight the minimum amount of time solutions should be left to react before measuring the absorbance, ensuring an efficient and robust assay. Overall, that this assay was able to detect differences between the meal fractions, demonstrates that the F–C reagent is appropriate for use in phenolic content determination assays in faba bean meal.

### Biocatalytic BLC23O-mediated reduction of phenolics in faba bean meal

**Fig. 4:**
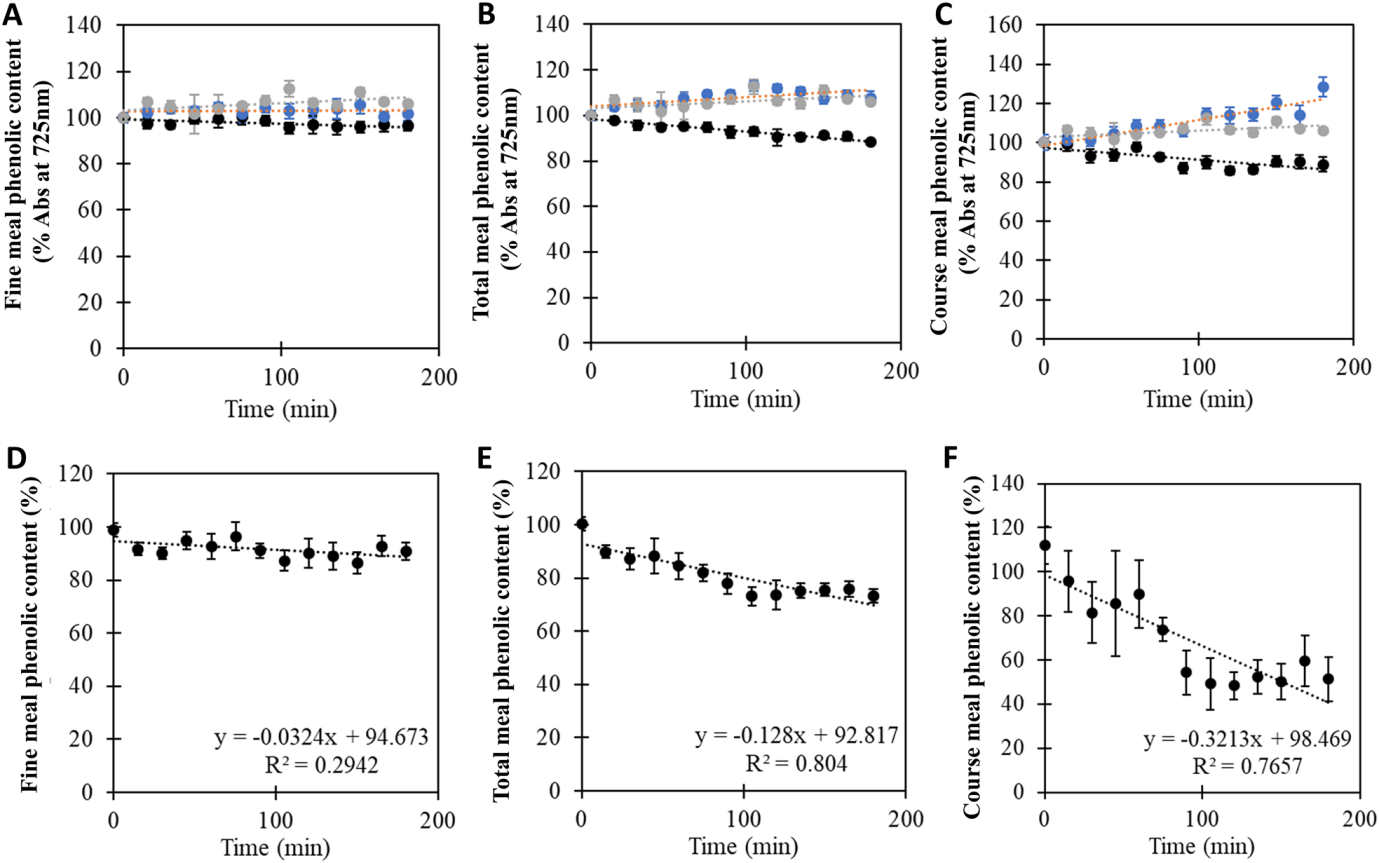
Biocatalytic reduction of phenols in faba bean meal using BLC23O. Phenolic content in faba bean total, fine and coarse meal fractions was evaluated in the presence (black) and absence (blue) of BLC23O over time. An enzyme-only control sample was also assessed (grey). Reactions between each meal type and BLC23O were initiated with the addition of 0.06 mg/mL of enzyme. Samples were taken in 15-min intervals and reacted with the Folin–Ciocalteu reagent. Time zero was set to 100% phenol and changes in absorbance at 725 nm plotted on a percentage change basis with **A** fine, **B** total and **C** coarse meal fractions. The observed changes in phenol content of BLC23O-treated **D** fine, **E** total and **F** coarse meal fractions were quantified relative to untreated fractions. The data represent the mean and standard deviation (*n* = 3). Linear fits of the data and the corresponding equations and coefficients of determination are shown

The ability of BLC23O to degrade phenols in total faba bean meal and the air classified fractions was evaluated. In all cases, BLC23O-treatment led to detectable decreases in phenol content over time, compared to untreated meal samples (Fig. 4A–C). Importantly, in both the meal-alone and enzyme-alone control samples, phenolic content remained constant or even displayed a slight increase over time. That increases in phenolic content were observed in the meal fractions (in the absence of any enzyme) is likely representative of the effect of chemical hydrolysis over time. Phenolic content can be inaccessible due to being embedded in large complex structures (Smeriglio et al. 2017; Soares et al. 2020). Water hydrolysis is known to release some phenols from the large complexes and break up interactions with proteins and sugars, increasing availability of smaller Mw phenolics to react with the enzyme and assay reagents. To account for this release, the ratio between phenolics in the BLC23O-treated samples and phenolics in the meal-only samples were determined. Additionally, background phenolics detected in the enzyme-only sample were subtracted. This analysis revealed a 49.6% decrease in phenolic content in the coarse meal fraction over 3 h (Fig. 4F). Phenolic content in the total meal was reduced by 26.7%, while only a 9.0% reduction was observed in the fine fraction (Fig. 4E and D, respectively). The coefficient of determination (R^2^) value for the linear regressions of the fine fraction type is low, reflecting the observed shallow slope, and highlighting the relatively low rate of phenolic reduction in this particular sample.

**Fig. 5:**
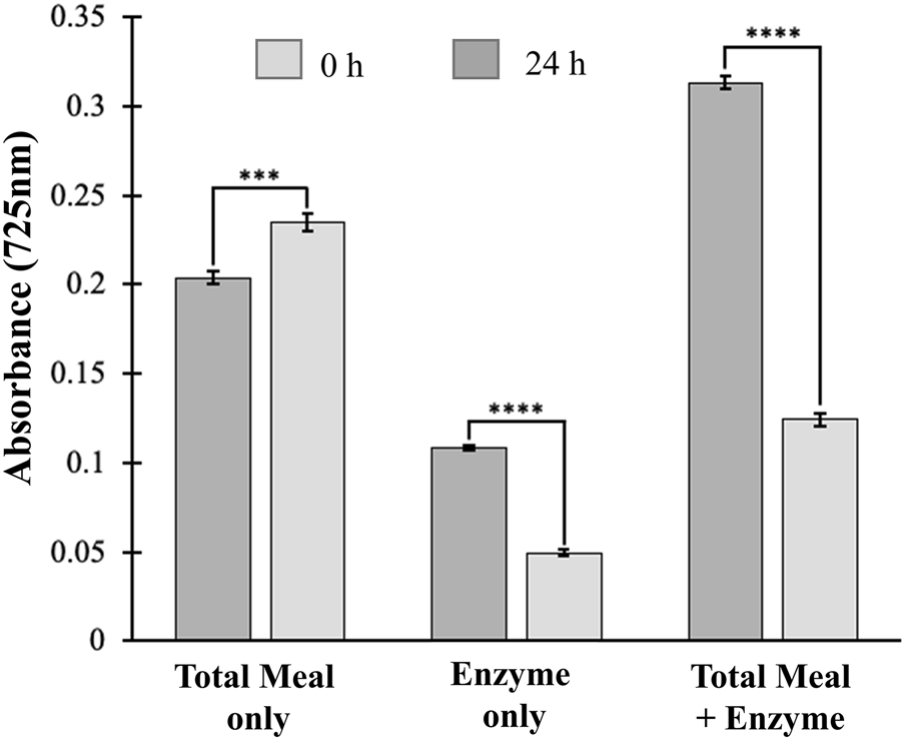
Effect of extended BLC23O reaction time on phenol content of faba bean meals. Change in total meal phenol content after 24 h reaction with BLC23O. 1.2 mg/mL of meal flour and 0.06 mg/mL of BLC23O were left to react at room temperature for 24 h and then reacted with the Folin–Ciocalteu reagent. Data are presented as the mean and standard deviation of the % absorbance at 725 nm (n = 3). ***p < 0.001, ****p < *0.0001*

To ascertain the stability of the reaction, overnight assays were performed for the total meal fraction in the presence and absence of BLC23O. Consistent with previous experiments, a small but significant increase in phenolics was detected in the meal-only sample, while decreases in both the enzyme-only and the BLC23O-treated meal samples were observed (Fig. 5). Quantification indicates a 68% decrease in phenolics in this meal fraction upon treatment with BLC23O for 24 h. This represents a 41% further decrease in phenolic content compared to values obtained after only 3 h, and emphasizes the catalytic stability of BLC23O over time in this complex environment.

### Biocatalytic BLC23O-mediated reduction of phenolic content in commercially available compounds

**Fig. 6:**
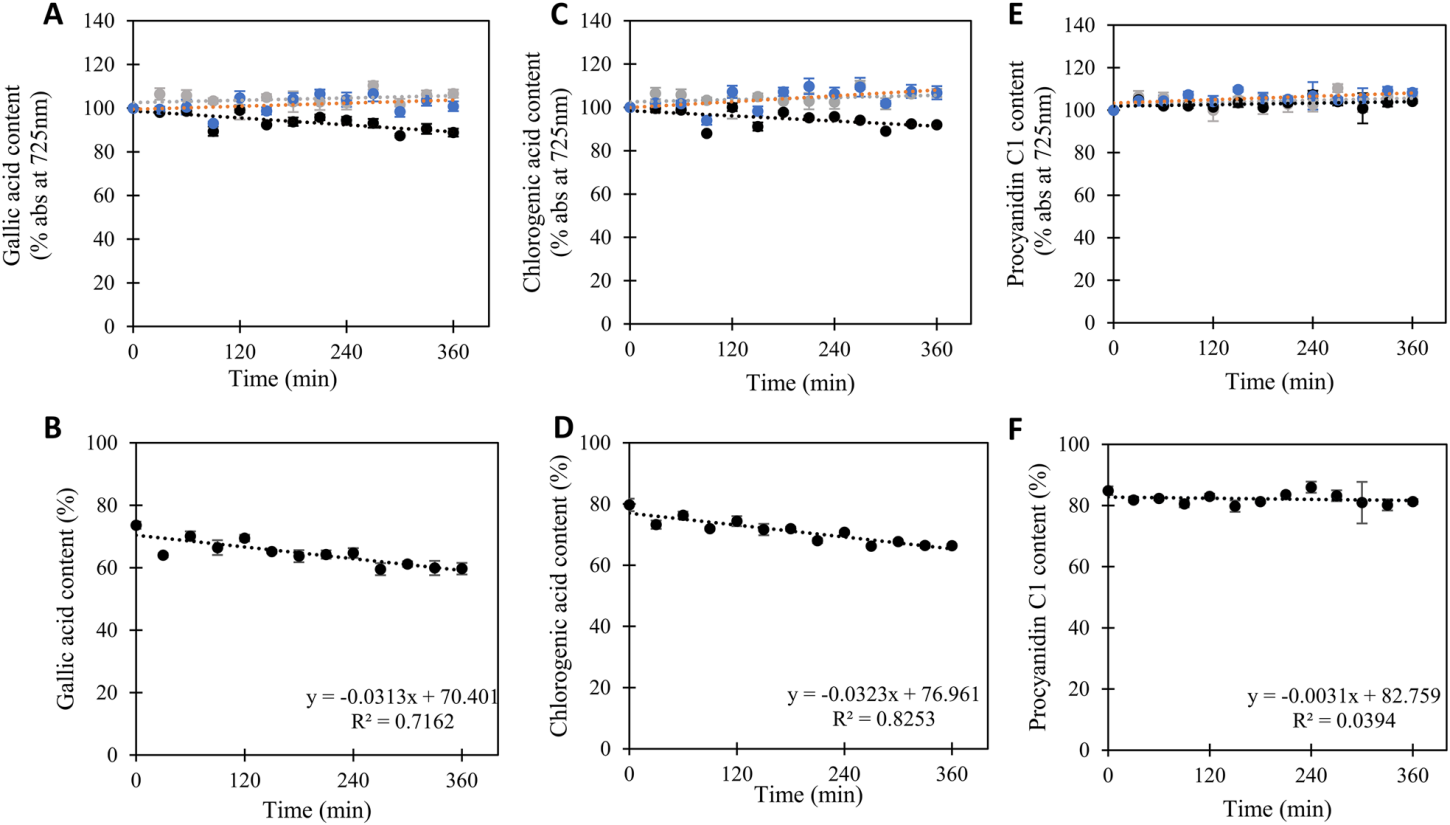
Biocatalytic reduction of phenols in pure phenolic compounds using BLC23O. Phenolic content in samples was evaluated in the presence (black) and absence (blue) of BLC23O over time. An enzyme-only control sample was also assessed (grey). Reactions were initiated with the addition enzyme at 32.5 °C. Samples were taken in 30 min intervals and reacted with the Folin–Ciocalteu reagent. Time zero was set to 100% phenol and changes in absorbance at 725 nm plotted on a percentage change basis for **A** gallic acid, **C** chlorogenic acid and **E** procyanidin C1. The observed changes in phenol content of BLC23O-treated **B** gallic acid, **D** chlorogenic acid and **F** procyanidin C1 were quantified relative to untreated fractions. The data represent the mean and standard deviation (*n* = 3). Linear fits of the data and the corresponding equations and coefficients of determination are shown. See also Additional file 1: Figure S4 for addition examples

To glean more insight into what phenolic compounds BLC23O might be addressing in the faba bean meal fractions, the ability of BLC23O to reduce the phenolic character of known phenolic standards was assessed. Standards were selected based on recent characterization of known phenolic compounds in faba bean meal (Baginsky et al. 2013; Elessawy et al. 2020; Johnson et al. 2021), and included gallic, chlorogenic and tannic acids, catechin, quercetin, polydatin, taxifolin and procyanidin C1 (Additional file 1: Figure S3). Application of BLC23O to mono-phenolics including gallic and chlorogenic acids yielded decreases of 14% and 13%, respectively, in phenolic content over the course of a 6 h incubation (Fig. 6 A-D). Notably, as observed for the faba bean meal, phenolic content of control reactions (enzyme only or substrate only) yielded no change or even a slight increase in phenolic content over time. In contrast, applications of BLC23O to larger Mw compounds had very little effect (Fig. 6 E, F; Additional file 1: Figure S4).

## Discussion

The focus of this study was faba bean, well known to contain an array of large and small Mw phenolic compounds (Baginsky et al. 2013; Elessawy et al. 2020; Johnson et al. 2021). Of particular note is the accumulation of flavan-3-ols including catechin, epicatechin and epigallocatechin along with some procyanidins. More broadly, faba bean also accumulates dihydromyricetin, taxifolin and polydatin (a glycoside of resveratrol) and somewhat uniquely chlorogenic acid (a glycosylated monophenolic). Assessment of the ability of BLC23O to address a cross section of these compounds as purified entities demonstrated the enzyme’s preference for hydroxylated mono-phenolic acids including chlorogenic acid (Fig. 6 and Additional file 1: Figures S3, S4), highlighting one likely target of the enzyme in the faba bean meal. At the same time, the limited activity of BLC23O against catechin, quercetin, polydatin taxifolin and procyanidin C1, emphasizes that a lot of the faba bean phenolic content is simply contained in larger Mw poly-phenolic compounds that are in themselves poor substrates for BLC23O.

Looking beyond faba bean, it is worth noting that different pulses contain significantly different profiles of phenolic compounds (Elessawy et al. 2020; Pathiraja et al. 2022). For example, common bean (*Phaseolus vulgaris* L.) somewhat uniquely contains high levels of the mono-phenolic ferulic acid, while dry pea (*Pisum sativum* L.) contains vanillic acid and chickpea (*Cicer arietinum* L.) contains 4-hydroxybenzoic acid. Lentil (*Lens culinaris* Medik.) also accumulates very high levels of vanillic acid, but primarily as a glycoside conjugate. Whether BLC23O would have activity against these mono-phenolics remains for future investigation, although the demonstrated activity against chlorogenic acid seems promising.

Although the profile of phenolics in the faba bean meal specifically used in this study was not obtained, the overall amount of phenolic content was assessed (Fig. 3). This included for the total meal, as well as two air classified fractions including the ‘fine’ protein enriched fraction and the ‘coarse’ carbohydrate enriched fraction (Coda et al. 2015). The observation of higher phenolics in the ‘fine’ fraction compared to the total meal sample is likely due to protein and phenolics binding tightly to form complexes, such that the phenolics are collected and concentrated with the protein. Interactions of poly-phenols with proteins are primarily dominated by hydrophobic interactions and hydrogen bonding (Amoako and Awika 2016; Asquith and Butler 1986; Frazier et al. 2003; Hagerman and Butler 1981; Hagerman et al. 1998). Some phenolic content was also observed in the ‘coarse’, carbohydrate-rich fraction which is consistent with recent evidence supporting strong and specific interactions between poly-phenols and carbohydrate polymers, also via hydrogen bonding and hydrophobic interactions. For example, the amylose component of starch forms non-digestible complexes with tannins likely due to some association between carbohydrates and phenolics (Amoako and Awika 2016; Barros et al. 2014; Kardum and Glibetic 2018; Smeriglio et al. 2017).

More broadly, a concerted search of the literature at the time of publication suggests there is no precedent for application of phenolic ring-breaking enzymes to bioprocessing of meals or feed in vitro. Indeed the primary industrial target of phenolic ring-breaking enzymes to date has been reduction of phenol-related pollution and toxins (Tian et al. 2017). This search also highlighted various enzymes more generally applied to food processing, and as additives to the diets of animals, but these also do not include phenolic ring-breaking enzymes (de Castro Leite Júnior et al. 2022). There is an obvious link between tannase and tannin degradation, but as mentioned previously, this activity has been accessed through fermentative efforts (der Poel et al. 2020; Olukomaiya et al. 2019). At the same time tannase only serves to degrade large Mw phenolics into smaller Mw phenolics (breaking ester bonds between the rings (Fig. 1A)) and does not strictly speaking contribute to reduction of phenolic content directly (Govindarajan et al. 2016). That said, the role of tannase in releasing mono-phenolics that can then be better addressed by ring-breaking enzymes, cannot be ignored. As well, that BLC23O prefers mono-phenolics, and cannot access the rings on large molecular weight poly-phenolics is clearly evident (Fig. 6 and Additional file 1: Figure S4). Thus, evaluation of the impact of pre-treatment of pulse meals with other enzymes like tannase, fermentation or physical processes to decrease the Mw of phenolic compounds to enable increased enzyme-based ring breaking remains for the future.

Finally, while the results highlighted here suggest that ring-breaking dioxygenases may be useful for reduction of mono-phenolics in feed and food and thus reduction of ANFs, one cannot ignore that there are many other types of ANFs present in meals. These include phytic acids, lectins, saponins, cyano- and sulfur-containing glycosides and allergenic proteins among others (Kumar et al. 2021). In the case of phytic acid, enzymatic strategies focused on application of phytase enzymes as a dietary supplement have been implemented (Bedford and Apajalahti 2022).

## Conclusions

In conclusion, this study demonstrates the viability of cell-free biocatalytic reduction of phenolic content in faba bean meal. The representative catechol dioxygenase, BLC23O, enzyme used in the proof-of-concept was selected based on its known stability and relatively broad substrate specificity. Its amenability to high yield fermentative recombinant production has now been established. Results suggest that BLC23O is effective for reduction of select small Mw ANF phenolic compounds in faba bean meal, in the absence of fermentation and with potential applications across other pulse crops. However, the upstream release of phenolics from higher molecular weight species including tannic acids, procyanidins, and quercetins (etc.) as well as protein and carbohydrate conjugates is likely a limiting factor in the absence of other enzymes or microbial fermentation. Analyses of the effect of including other enzymes with BLC23O to improve substrate availability, and the effect of adding BLC23O to fermentative processes is ongoing, toward optimization of the nutritional and economical value of pulse meal in the animal feed industry.

## Methods and materials

All chemicals were obtained from Sigma Aldrich, except where indicated below. Faba beans were obtained from Faba Canada Ltd, de-hulled at the Canadian International Grains Institute (Winnipeg, Canada), and milled/air classified at the Richardson Center for Food Technology and Research (Winnipeg, Canada), yielding a ‘total’ meal fraction (before air classification), and then air classification-derived ‘fine’ and ‘coarse’ meal fractions.

### BLC23O fermentative scale production

An optimized version of the BLC23O coding region was cloned into the pET28B + vector such that the expressed fusion protein included an N-terminal 6 × His tag as previously described (Adewale et al. 2021). *Escherichia coli* BL21 cells containing the expression vector encoding the BLC23O gene was used to inoculate an overnight culture containing 50 mL of Terrific Broth (TB) medium (24 g/L yeast extract, 20 g/L tryptone, 4 mL/L glycerol, 0.017 M KH_2_PO_4_, 0.072 M K_2_HPO_4_) with 50 µg/mL kanamycin. The culture was incubated overnight at 37 °C in a MaxQ 6000 shaker (Thermo Scientific) at 150 rpm. The overnight culture was used to inoculate a BioFlo/CelliGen 115 benchtop fermentor & bioreactor (New Brunswick Scientific) containing 4 L of TB medium, 50 mg/L kanamycin, and 0.5 mL of antifoam. The bioreactor had an agitation of 250 rpm, its water jacket temperature was set to 37 °C, and the dissolved oxygen setpoint was 30%. The culture was grown to an OD600 between 0.6 and 0.7 at which time protein expression was induced with the addition of 0.7 mM isopropyl β-D-1-thiogalactopyranoside (IPTG). The culture was left growing in the bioreactor overnight at 18 °C. The media was centrifuged in a Sorvall Lynx 4000 centrifuge (Thermo scientific) at 3250 × g for 30 min at 4 °C.

### Purification of recombinantly produced BLC23O

The obtained pellet was resuspended in 500 mL of 1 × lysis buffer (50 mM NaH_2_PO_4_, 300 mM NaCl, 10 mM imidazole, 0.1 mM phenylmethylsulfonyl fluoride (PMSF), 3U/mL benzonase, 1 mg/mL lysozyme, pH 8.0). Proteins were extracted by high pressure homogenization using an EmulsiFlex-C5 (Avestin) according to the manufacturer’s protocol. The lysate was centrifuged (Thermo scientific) at 12,000 rpm for 15 min at 4 °C. The supernatant was collected and applied to nickel–nitrilotriacetic acid (Ni–NTA) resin (Qiagen) with binding buffer (50 mM NaH_2_PO_4_, 300 mM NaCl, 10 mM imidazole, pH 8.0) by a batch method, where resin was mixed with the cleared lysate in a MaxQ 6000 shaker (Thermo Scientific) set to 200 rpm at 4 °C for 2 h. The sample was centrifuged in a Sorvall Legend X1R centrifuge at 1000 × g for 3 min at 4 °C (Thermo scientific) and the supernatant discarded. The remaining beads were washed three times using this same process with wash buffer (50 mM NaH_2_PO_4_, 300 mM NaCl, 20 mM imidazole, pH 8.0), and then BLC23O was eluted using elution buffer (50 mM NaH_2_PO_4_, 300 mM NaCl, 250 mM imidazole, pH 8.0). Obtained elution supernatants containing the BLC23O enzymes were pooled and concentrated using an Amicon Ultra-15 centrifugal filter with a 10 kDa cutoff (MilliporeSigma). The final concentration of the obtained BLC23O sample was determined using a Quick Start Bradford protein assay (Bio-Rad) according to the manufacturer’s protocol. Proteins samples were visualized by 12% SDS-PAGE.

A small portion (approx. 4% of the total sample) of the obtained enriched enzyme was further subjected to Fast Performance Liquid Chromatography (FPLC; Amersham Pharmacia Biotech) size-exclusion chromatography (SEC) with a HiLoad 16/600 Superdex 200 (Cytvia) column. Size exclusion buffer (10 mM Tris–HCl, 150 mM NaCl, pH 7.4) was degassed and the FPLC flowrate was set to 1 mL/min. Elution fractions were collected in increments of 1.4 mL. Fractions containing BLC23O were combined and concentrated with an Amicon Ultra-15 centrifugal filter with a 10 kDa cutoff (MilliporeSigma). The final concentrations of BLC23O were determined using a Pierce bicinchoninic acid (BCA) protein assay kit (Thermo Scientific) according to the manufacturer’s protocol. Proteins samples were visualized by 12% SDS-PAGE.

### BLC23O kinetic analysis

Reaction solutions with total volumes of 250 µL were prepared in triplicate in a 96-well microplate (Greiner Bio-One) with 50 mM phosphate buffer pH 7.5, 18.7 µg/mL of BLC23O purified by Ni–NTA and SEC, 10 mM Mn^2+^, and substrate (3-methylcatechol) concentrations varying from 0.05 mM to 3 mM. Plates were pre-incubated for 5 min at 32.5 °C and reactions were initiated with the addition of substrate and samples left at 32.5 °C. The absorbance was read at 388 nm for 60 min in 30 s intervals using a SpectraMax M5e spectrophotometer (Molecular Devices). The initial reaction rate, in µM of product formed per second, was plotted and the data fitted to the Michaelis–Menten equation using Microsoft Excel’s Solver add-in.

### Determination of phenolic content by F–C reagent

Based on previous reports (Blainski et al. 2013; Everette et al. 2010), a calibration curve for the quantification of phenolic content was created where reaction solutions with a volume of 1 mL were prepared with 0.125 N F–C reagent, 0.125 g/mL of sodium carbonate decahydrate, and tannic acid concentrations ranging from 0 to 30 µg/mL. The solutions were left to react for 45 min and then 250 µL of each was transferred into a 96-well microplate (Greiner Bio-One) and the absorbance was read at 725 nm using a SpectraMax M5e spectrophotometer (Molecular Devices). This was repeated in triplicate. Tannic acid reaction kinetics were assessed in a similar manner, but with reaction solution volumes of 250 µL and tannic acid concentrations ranging from 0 to 14 µg/mL. In this case, as soon as the tannic acid was added, the absorbance at 725 nm was monitored for 180 min in 15-min intervals using an Epoch 2 microplate spectrophotometer (Biotek).

Initial phenolic content in the three faba bean meal fractions was determined using the same procedure as described above, but with 0.1, 0.2, and 0.3 mg/mL of each type of faba bean sample instead of tannic acid, and incubation with the F–C reagent for 60 min. Results were compared to the tannic acid calibration curve for quantification, based on the assumption that a µg of commercial tannic acid is equivalent to a µg of phenolic content in the meal.

### BLC23O-mediated phenolic reduction in faba bean flour and purified compounds

BLC23O reaction solutions with volumes of 4 mL were prepared for each of the faba bean meal fractions with 1.2 mg/mL of each fraction type and reactions initiated with the addition of 0.06 mg/mL of BLC23O enzyme. A blank solution with no meal or enzyme, as well negative and background controls of meal only and enzyme only were also prepared. Samples from the reaction mixtures were taken at 15-min intervals for 3 h, vortexed into 0.125 N F–C reagent and 0.125 g/mL of sodium carbonate decahydrate and then left to react for 60 min at room temperature. Subsequently, 250 µL from each mixture was transferred to a 96-well microplate (Greiner Bio-One) and the absorbance was read at 725 nm using Epoch 2 microplate spectrophotometer (Biotek). This was performed in triplicate. The % phenolic reduction was calculated using the following equation: (phenolic content of meal in reaction mixture (%) = (R_abs (t)—E_abs (t)) / (F_abs (t)) * 100%, where Rabs(t) is the absorbance of the reaction mixture at time t, Eabs(t) is the absorbance of the enzyme sample at time t, and Fabs(t) is the absorbance of the specific flour (fine, meal or coarse) at time t. Additional faba bean meal assays were left to react for 24 h before being added to the F–C and sodium carbonate.

In the case of commercially available compounds, each target compound was dissolved in a suitable solvent and at a concentration to achieve an absorbance reading in the range of 0.2 to 0.3 OD at 725 nm (Table 1; determined in the absence of enzyme), and treated as described above.

**Table 1:** List of commercially available phenolic compounds tested in this study

Compound	Concentration (µM)	Solvent
Gallic acid	50	Water
Chlorogenic acid	35	Water
Procyanidin C1	15	Water
Tannic acid	5	Water
( +)-Catechin	15	Water
Quercetin	15	Water, 1% tween 40
Taxifolin	15	Water, 0.25% methanol
Polydatin (resveratrol-3-β-mono-d-glucoside)	50	Water, 0.8% methanol

### Statistical analysis and graphing

Statistical analyses and graphing of data were performed using Microsoft Excel. Unless otherwise stated, statistical analyses were done using a t-test and a p-value of 0.05 or lower was considered significant.

## Supplementary Information

**Additional file 1: Figure S1.** Purification of BLC23O by size-exclusion chromatography. A) Size-exclusion chromatography (SEC) was performed with a HiLoad16/600 Superdex200 column attached to an FPLC system and run at a flowrate of 1 mL/min. A) The elution profile (OD280nm) with the ladder at the bottom of the graph representing the fractions collected during the run. B)Elution profile for standard proteins to calibrate the SEC column where F is ferritin, Ald is aldolase, C is conalbumin, O is ovalbumin (45kDa), CA is carbonic anhydrase (29.2 kDa), R is RNase A, and Apr is aprotinin (Cytvia). C)A 12% acrylamide SDS-PAGE analysis of select fractions from the BLC23O SEC purification. **Figure S2.** Folin–Ciocalteu assay development. A) Calibration curve for determination of total phenolic content. Folin–Ciocalteu reagent was added to known concentrations of tannic acid ranging from 0 to 30 ug/mL and left to react for 45 min. Absorbances were then measured at 725 nm. The data represent the mean and standard deviation (n=3). A linear fit of the data with an equation of y=0.0127x and a R^2^ value of 0.9981 are shown. B) Evaluation of Folin–Ciocalteu reagent kinetics. Reaction solutions with volumes of 250 μL were prepared with 0.125 N Folin–Ciocalteu reagent, 0.125 mg/mL sodium carbonate, and 14 μg/mL tannic acid. The absorbance at 725 nm was monitored for 180 min in 15-min intervals. The data represent the mean and standard deviation (n=3). **Figure S3.** Representative images of commercially available phenolic compounds tested as substrates for BLC23O. **Figure S4.** Biocatalytic reduction of phenols in purified phenolic compounds using BLC23O. Top row) Phenolic content in samples containing purified compounds as indicated, was evaluated in the presence (black) and absence (blue) of BLC23O over time. An enzyme-only control sample was also assessed (grey). Reactions were initiated with the addition enzyme at 32.5 °C. Samples were taken in 30 min intervals and reacted with the Folin–Ciocalteu reagent. Time zero was set to 100 % phenol and changes in absorbance at 725nm plotted on a percentage change basis. Bottom row) The observed changes in phenol content of BLC23O treated compounds as indicated were quantified relative to untreated fractions. The data represent the mean and standard deviation (n=3). Linear fits of the data and the corresponding equations and coefficients of determination are shown.

## Data Availability

All data generated or analysed during this study are included in this published article [and its Additional files].

## Abbreviations

BLC23O: *Bacillus ligniniphilus* L1 catechol 2,3-dioxygenase
ANFs: Anti-nutritional factors
F–C: Folin–Ciocalteu
