# Supplementary material for: Reduction of phenolics in faba bean meal using recombinantly produced and purified Bacillus ligniniphilus catechol 2,3-dioxygenase
Source: Bioresour Bioprocess. 2023 Feb 12;10(1):13. doi: 10.1186/s40643-023-00633-8 (PMC9925492; doi:10.1186/s40643-023-00633-8)
Supplement: Supplementary file 1 — Additional file 1: Figure S1. Purification of BLC23O by size-exclusion chromatography. A) Size-exclusion chromatography (SEC) was performed with a HiLoad16/600 Superdex200 column attached to an FPLC system and run at a flowrate of 1 mL/min. A) The elution profile (OD280nm) with the ladder at the bottom of the graph representing the fractions collected during the run. B)Elution profile for standard proteins to calibrate the SEC column where F is ferritin, Ald is aldolase, C is conalbumin, O is ovalbumin (45kDa), CA is carbonic anhydrase (29.2 kDa), R is RNase A, and Apr is aprotinin (Cytvia). C)A 12% acrylamide SDS-PAGE analysis of select fractions from the BLC23O SEC purification. Figure S2. Folin–Ciocalteu assay development. A) Calibration curve for determination of total phenolic content. Folin–Ciocalteu reagent was added to known concentrations of tannic acid ranging from 0 to 30 ug/mL and left to react for 45 min. Absorbances were then measured at 725 nm. The data represent the mean and standard deviation (n=3). A linear fit of the data with an equation of y=0.0127x and a R2 value of 0.9981 are shown. B) Evaluation of Folin–Ciocalteu reagent kinetics. Reaction solutions with volumes of 250 μL were prepared with 0.125 N Folin–Ciocalteu reagent, 0.125 mg/mL sodium carbonate, and 14 μg/mL tannic acid. The absorbance at 725 nm was monitored for 180 min in 15-min intervals. The data represent the mean and standard deviation (n=3). Figure S3. Representative images of commercially available phenolic compounds tested as substrates for BLC23O. Figure S4. Biocatalytic reduction of phenols in purified phenolic compounds using BLC23O. Top row) Phenolic content in samples containing purified compounds as indicated, was evaluated in the presence (black) and absence (blue) of BLC23O over time. An enzyme-only control sample was also assessed (grey). Reactions were initiated with the addition enzyme at 32.5 °C. Samples were taken in 30 min intervals and reacted with the Folin–Ci [file 40643_2023_633_MOESM1_ESM.pdf]

Reduction of phenolics in faba bean meal using recombinantly produced and purified *Bacillus ligniniphilus* catechol 2,3-dioxygenase

Rebecca M. Murphy[1], Joanna C. Stanczyk[2], Fang Huang[2], Matthew E. Loewen[3], Trent C. Yang[2], Michele C. Loewen[1,2,*]

[1] Department of Chemistry and Biomolecular Sciences, University of Ottawa, 150 Louis-Pasteur Pvt, Ottawa, ON, K1N 6N5, Canada
[2] Aquatic and Crop Resources Development Research Center, National Research Council of Canada, 100 Sussex Drive, Ottawa, Ontario, K1A 0R6, Canada.
[3] Department of Veterinary Biomedical Sciences, Western College of Veterinary Medicine, University of Saskatchewan, 52 Campus Drive, Saskatoon, Saskatchewan, S7N 5B4, Canada

*Corresponding author: michele.loewen@nrc.ca; National Research Council of Canada, 100 Sussex Drive, Ottawa, Ontario, K1A 0R6, Canada.

Additional file Figures

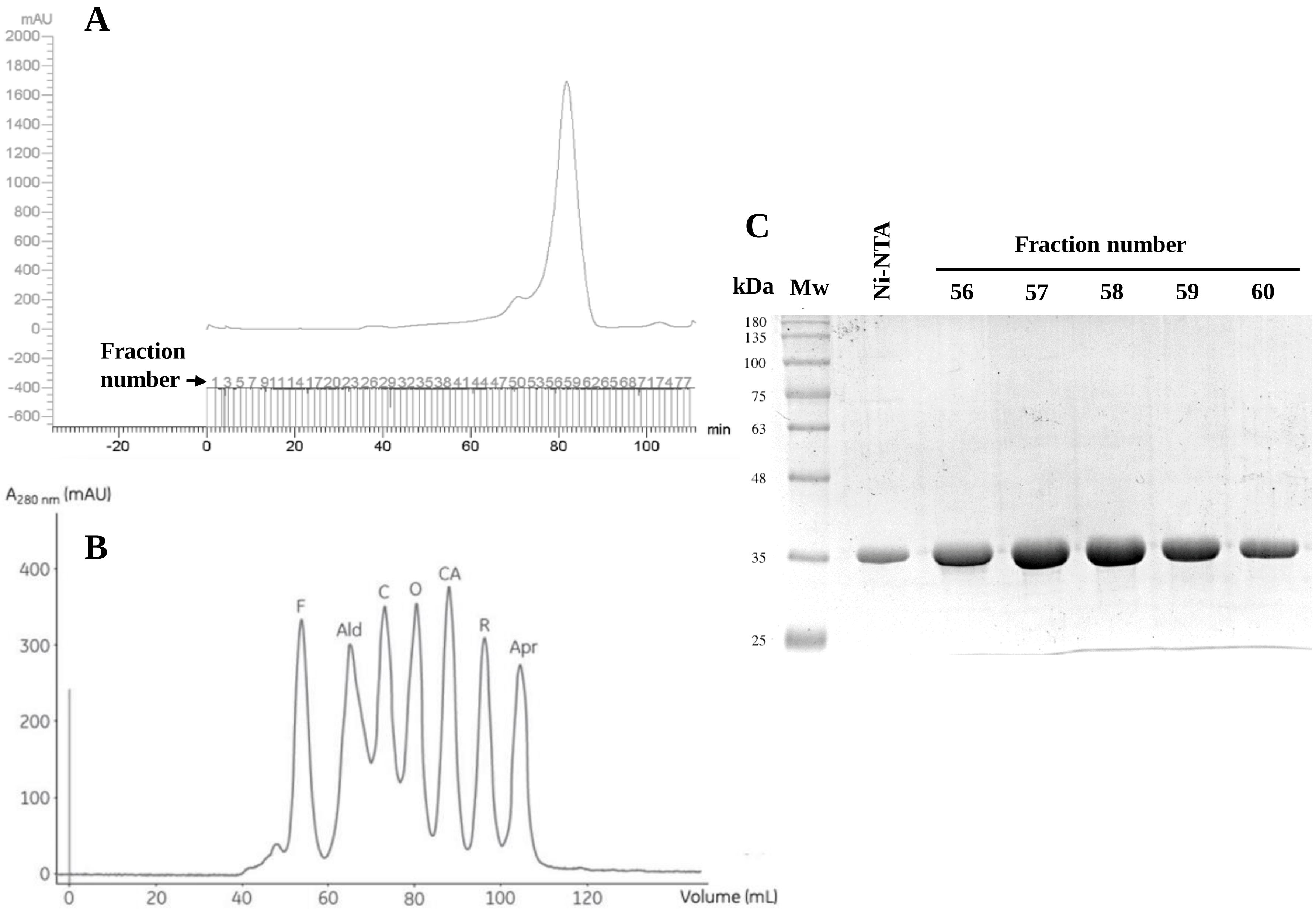

Figure S1:
Purification of BLC23O by size-exclusion chromatography. A) Size-exclusion chromatography (SEC) was performed with a HiLoad 16/600 Superdex 200 column attached to an FPLC system and run at a flowrate of 1 mL/min. A) The elution profile (OD280nm) with the ladder at the bottom of the graph representing the fractions collected during the run. **B)** Elution profile for standard proteins to calibrate the SEC column where F is ferritin, Ald is aldolase, C is conalbumin, O is ovalbumin (45kDa), CA is carbonic anhydrase (29.2 kDa), R is RNase A, and Apr is aprotinin (Cytvio). **C)** A 12% acrylamide SDS-PAGE analysis of select fractions from the BLC23O SEC purification..

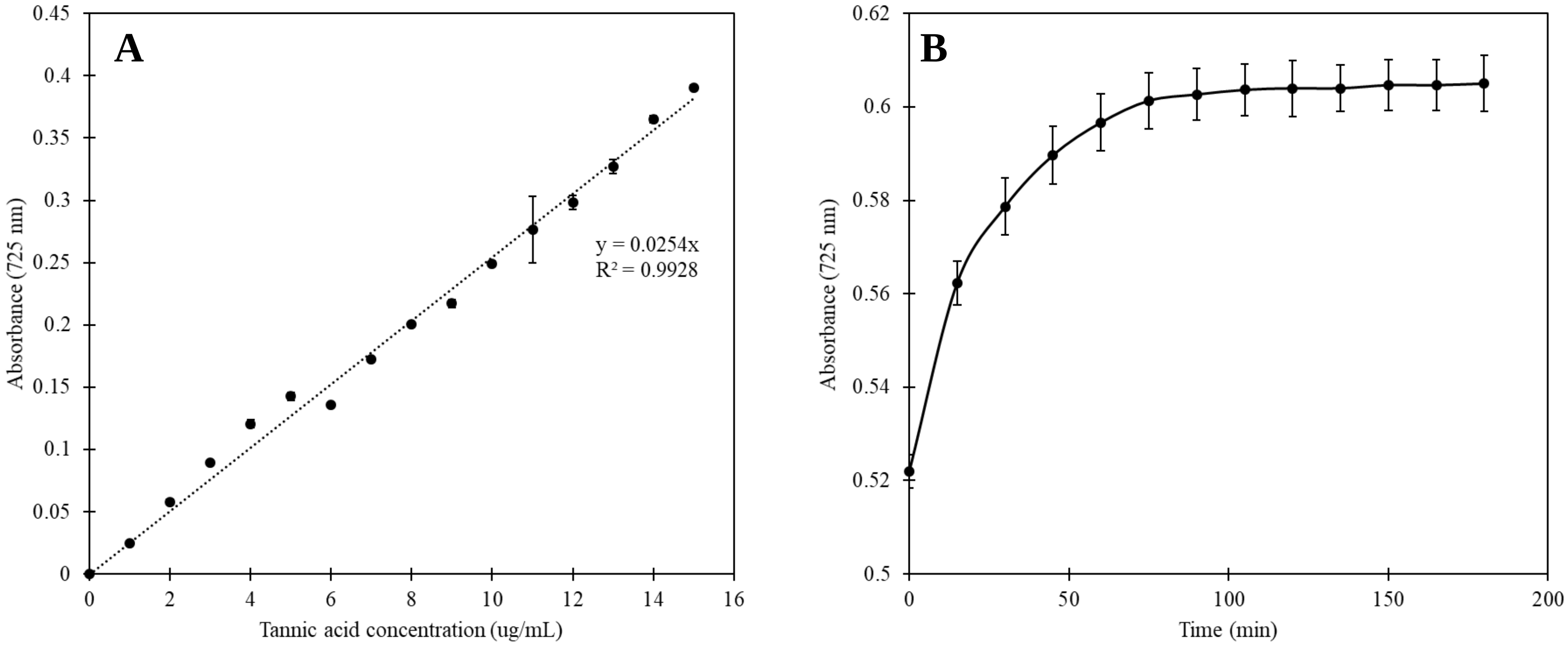

Figure S2: Folin-Ciacalteu assay development. A) Calibration curve for determination of total phenolic content. Folin-Ciocalteu reagent was added to known concentrations of tannic acid ranging from 0 to 30 ug/mL and left to react for 45 min. Absorbances were then measured at 725 nm. The data represents the mean and standard deviation (n=3). A linear fit of the data with an equation of y=0.0127x and a R2 value of 0.9981 are shown. **B)** Evaluation of Folin-Ciocalteu reagent kinetics. Reaction solutions with volumes of 250 µL were prepared with 0.125 N Folin-Ciocalteu reagent, 0.125 mg/mL sodium carbonate, and 14 µg/mL tannic acid. The absorbance at 725 nm was monitored for 180 min in 15 min intervals. The data represents the mean and standard deviation (n=3).

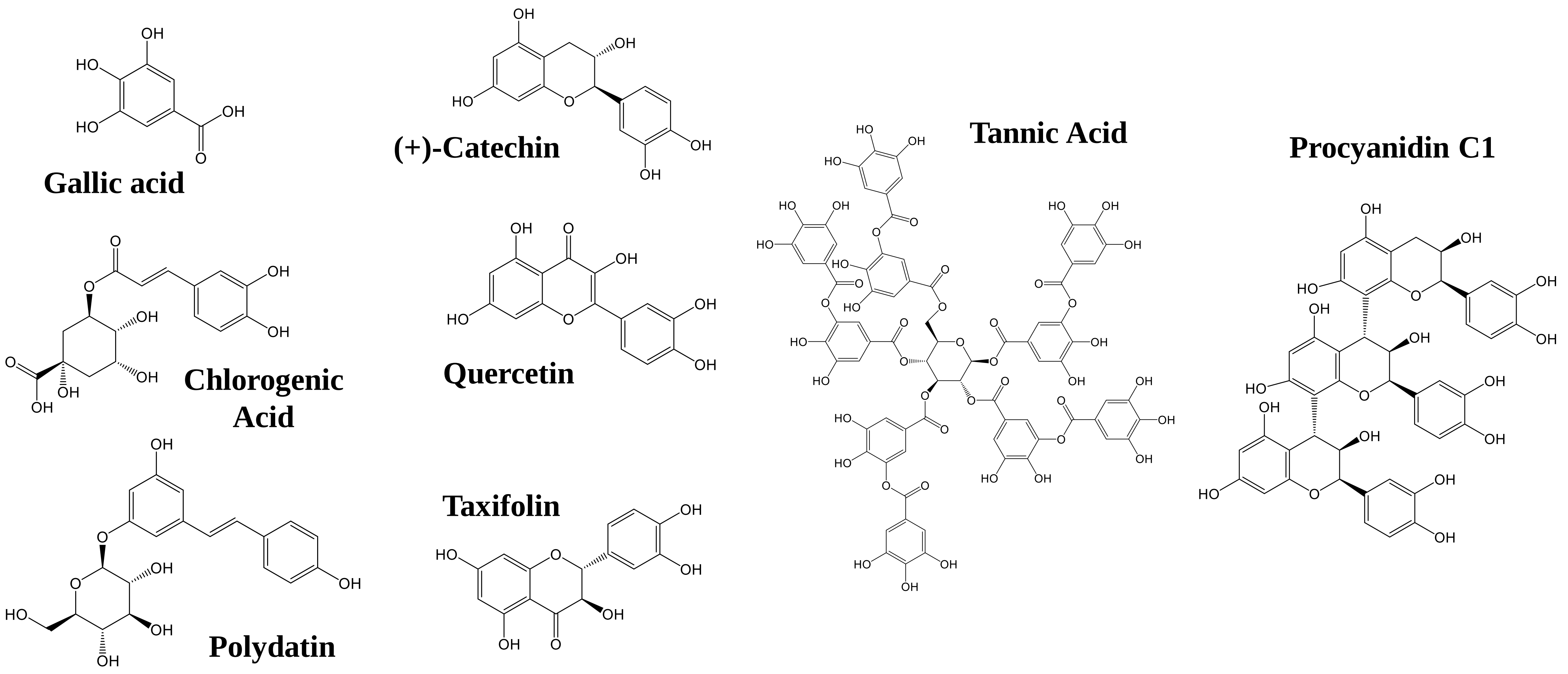

Figure S3: Representative images of commercially available phenolic compounds tested as substrates for BLC23O.

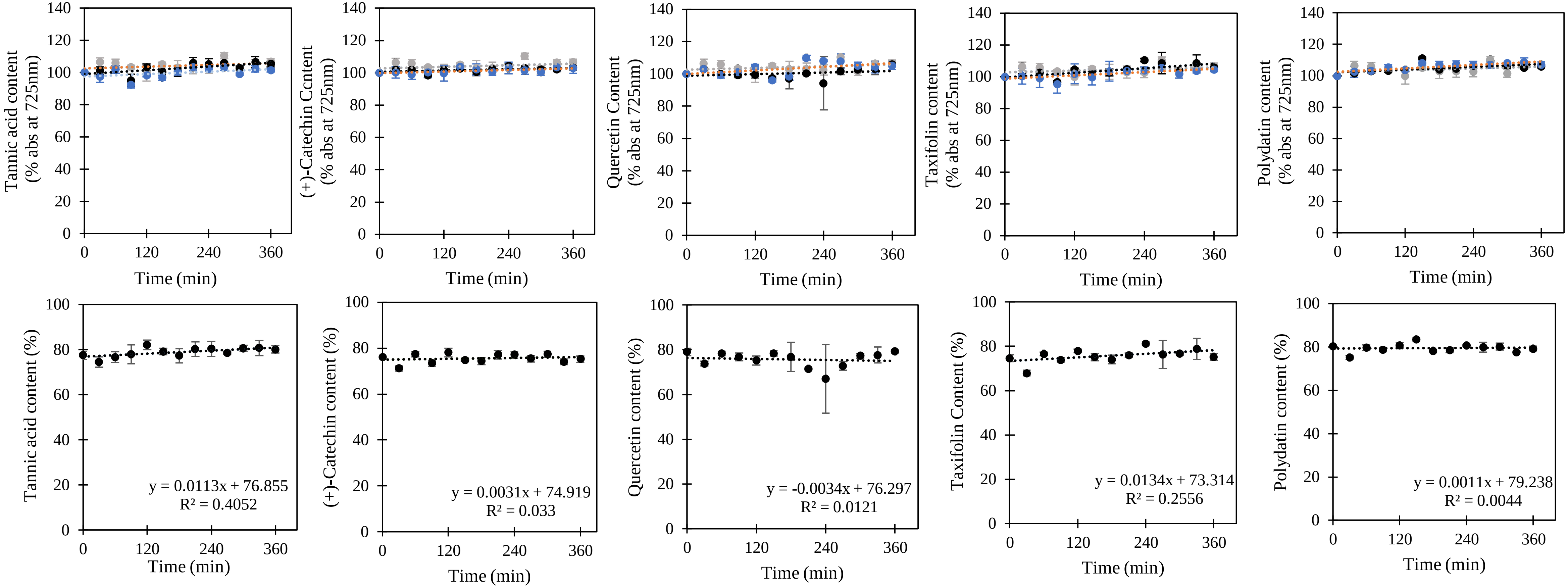

Figure S4: Biocatalytic reduction of phenols in purified phenolic compounds using BLC23O. Top row) Phenolic content in samples containing purified compounds as indicated, was evaluated in the presence (black) and absence (blue) of BLC23O over time. An enzyme only control sample was also assessed (grey). Reactions were initiated with the addition enzyme at 32.5 °C. Samples were taken in 30 min intervals and reacted with the Folin-Ciocalteu reagent. Time zero was set to 100 % phenol and changes in absorbance at 725nm plotted on a percentage change basis. **Bottom row)** The observed changes in phenol content of BLC23O treated ompunds as indicated were quantified relative to untreated fractions. The data represents the mean and standard deviation (n=3). Linear fits of the data and the corresponding equations and coefficients of determination are shown.
